# Supplementary material for: Histological analysis of post-eruption tooth wear adaptations, and ontogenetic changes in tooth implantation in the acrodontan squamate Pogona vitticeps
Source: PeerJ. 2018 Nov 8;6:e5923. doi: 10.7717/peerj.5923 (PMC6230436; doi:10.7717/peerj.5923)
Supplement: Table S1 [file peerj-06-5923-s001.docx]

| ROM# | Family | Genus | Species | # of teeth | SEX |  |  |
| --- | --- | --- | --- | --- | --- | --- | --- |
| 5946 | Agamidae | Pogona | vitticeps |  | F | Skeleton; complete; | WT= 459G; TL= 45.4CM; SV= 20.5CM; 'SHIRAZ' |
| 7203 | Agamidae | Pogona | vitticeps |  | M | Skeleton; complete; | WT= 323G; TL= 46CM; SV= 21CM; LIVER TUMOURS |
| 7205 | Agamidae | Pogona | vitticeps |  | F | Skeleton; complete; | WT= 179G; TL= 38CM; SV= 17.5CM |
| 7400 | Agamidae | Pogona | vitticeps |  | F | Skeleton; complete; | WT= 511GR; TL= 52CM; SV= 23.3CM; PHOTOGRAPHED |
| 7472 | Agamidae | Pogona | vitticeps |  | M | Skeleton; complete; | WT= 70G; TL= 34.5CM; SV= 14.2CM; PHOTOGRAPHED |
| 7473 | Agamidae | Pogona | vitticeps |  | M | Skeleton; complete; | WT= 216G; TL= 44CM; SV= 21.6CM; PHOTOGRAPHED; DEHYDRATED, STUBBED TAIL |
| 7474 | Agamidae | Pogona | vitticeps |  | F | Skeleton; complete; | WT= 203G; TL= 30.3CM; SV= 19.3CM; PHOTOGRAPHED, STUBBED TAIL |
| 7476 | Agamidae | Pogona | vitticeps |  | F | Skeleton; complete; | WT= 347G; TL= 34CM; SV= 18.7CM; PHOTOGRAPHED, STUBBED TAIL |
| 7477 | Agamidae | Pogona | vitticeps |  | F | Skeleton; complete; | WT= 271G; TL= 21.7CM; SV= 30CM; PHOTOGRAPHED, STUBBED TAIL |
| 8105 | Agamidae | Pogona | vitticeps | 20 | M | Skeleton; complete; | WT= 349g; TL= 49.5cm; SV= 22cm |
| 8144 | Agamidae | Pogona | vitticeps | ?? | F | Skeleton; complete; | WT= 25g; TL= 35.2cm; SV= 21.9cm |
| 8189 | Agamidae | Pogona | vitticeps | 19 | M | Skeleton; complete; | WT= 449g; TL= 50.2cm; SV= 21.1cm |
| 8190 | Agamidae | Pogona | vitticeps | 18 | F | Skeleton; complete; | WT= 237g; TL= 46.4cm; SV= 21.4cm |
| 8226 | Agamidae | Pogona | vitticeps | 10 | F | Skeleton; complete; | WT= 232 g; TL= 42.8 cm; SV= 19.1 cm; photographed |
| 8227 | Agamidae | Pogona | vitticeps | 18 | F | Skeleton; complete; | WT= 245 g; TL= 38.3 cm; SV= 19.3 cm; photographed |
| 8228 | Agamidae | Pogona | vitticeps | 15 | M | Skeleton; complete; | WT= 360 g; TL= 46.2 cm; SV= 26.7 cm; photographed |
| 8229 | Agamidae | Pogona | vitticeps |  | M | Skeleton; complete; | WT= 332 g; TL= 41.1 cm; SV= 22.3 cm; photographed |
| 8234 | Agamidae | Pogona | vitticeps | 16 | M? | Skeleton; complete; juvenile; | WT= 27.7 g; TL= 23.8 cm; SV= 10.3 cm; photographed |
| 8412 | Agamidae | Pogona | vitticeps | 13 |  | Skeleton; complete; juvenile; | WT= 9.0g; TL= 18.6cm; SV= 7.6cm |
| 8413 | Agamidae | Pogona | vitticeps |  |  | Skeleton; complete; juvenile; | WT= 9.9g; TL= 15.7cm; SV= 6.3cm |
| 8414 | Agamidae | Pogona | vitticeps | 12 |  | Skeleton; complete; juvenile; | WT= 4.5g; TL= 15.8cm; SV= 6.3cm |
| 8415 | Agamidae | Pogona | vitticeps |  |  | Skeleton; complete; juvenile; | WT= 1.4g; TL= 5.6cm lacking tip of tail; SV= 4.1cm |
| 8416 | Agamidae | Pogona | vitticeps |  |  | Skeleton partial; juvenile; | WT= 1.5g lacking forelimbs; TL= 7.8cm; SV= 4.2cm |
| 8417 | Agamidae | Pogona | vitticeps |  |  | Skeleton partial; juvenile; | WT= 1.1g lacking forelimbs; TL= 7.8cm; SV= 4.1cm |
| 8418 | Agamidae | Pogona | vitticeps |  |  | Skeleton; complete; juvenile; | WT= 1.9g lacking forelimbs; TL= 6.4cm; SV= 4.4cm |
| 8419 | Agamidae | Pogona | vitticeps |  |  | Skeleton; complete; juvenile; | WT= 1.2g; TL= 6.8cm; SV= 3.8cm |
| 8420 | Agamidae | Pogona | vitticeps | 10 |  | Skeleton; complete; juvenile; | WT= 1.8g; TL= 9.5cm; SV= 4.4cm |
| 8504 | Agamidae | Pogona | vitticeps | 15 | M | Skeleton; complete; | WT= 324g; TL= 21.9cm; SV= 47.2cm |
| 8505 | Agamidae | Pogona | vitticeps | 17 | M | Skeleton; complete; | WT= 372g; TL= 21.8cm lacking tip of tail; SV= 42.0+cm |
| 8506 | Agamidae | Pogona | vitticeps | 17 | M | Skeleton; complete; | WT= 292g; TL= 44.1cm; SV= 19.5cm |
| 8507 | Agamidae | Pogona | vitticeps | 18? | M | Skeleton; complete; | WT= 441g; TL= 48.4cm; SV= 22.2cm |
| 8508 | Agamidae | Pogona | vitticeps | ?? | M | Skeleton; complete; | WT= 236g; TL= 44.6+cm lacking tip of tail; SV= 19.1cm |
| 8510 | Agamidae | Pogona | vitticeps | 18 | F | Skeleton; complete; | WT= 152g; TL= 39.7cm; SV= 17.8cm |
| 8514 | Agamidae | Pogona | vitticeps | 17 | F | Skeleton; complete; | WT= 229g; TL= 41.9cm; SV= 19.7cm |
| 8515 | Agamidae | Pogona | vitticeps |  | M | Skeleton; complete; | WT= 158+g; TL= N/A; SV= 17.2cm; lacking tail |
| 9075 | Agamidae | Pogona | vitticeps |  | M | Skeleton; complete; | WT= 151.4 g; TL= 43.5 cm; SV= 19.2 cm; skull cut for endocast |
| 9118 | Agamidae | Pogona | vitticeps |  |  | Skeleton; complete; | WT= 39.7 g; TL= 26.3 cm; SV= 11.1 cm |
| 9422 | Agamidae | Pogona | vitticeps |  | M | Skeleton; complete; | WT= 280.3 g; TL= 42.2; SV= 18.1 cm |
