# Supplementary figures and images for: Histological analysis of post-eruption tooth wear adaptations, and ontogenetic changes in tooth implantation in the acrodontan squamate Pogona vitticeps
Source: PeerJ. 2018 Nov 8;6:e5923. doi: 10.7717/peerj.5923 (PMC6230436; doi:10.7717/peerj.5923)

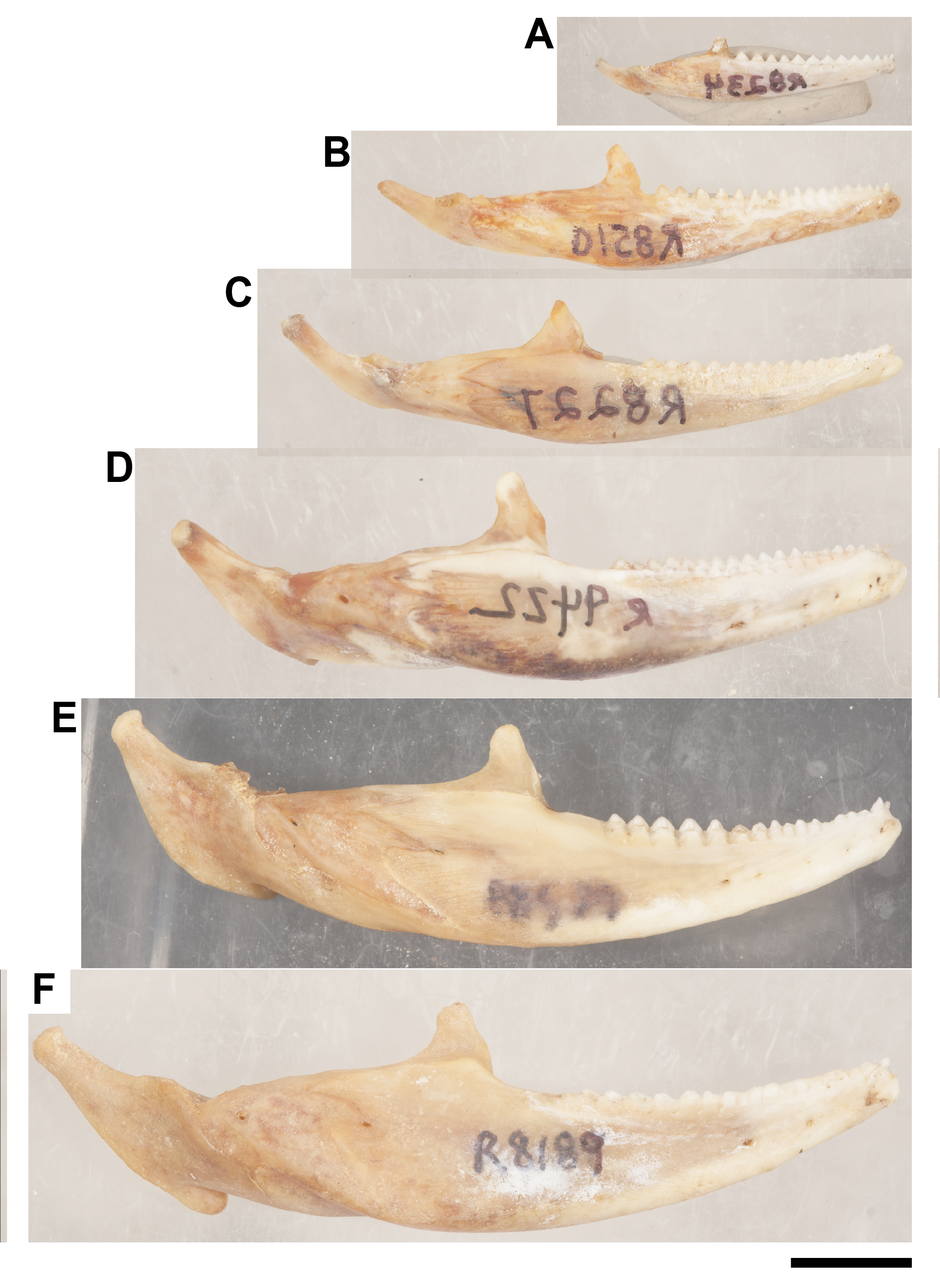

Supplement: Figure S1 — (A) ROM R8234; (B) ROM R8150; (C) ROM R8227; (D) ROM R9422; (E) ROM R8507; (F) ROM R8189. Scale bar = 1 cm. Photo credit: Diane Scott. [file peerj-06-5923-s002.png]
